# Enhancing Biomedicine: Proteomics and Metabolomics in Action

**DOI:** 10.3390/proteomes13010005

**Published:** 2025-01-16

**Authors:** Michele Costanzo, Marianna Caterino, Lucia Santorelli

**Affiliations:** 1 Department of Molecular Medicine and Medical Biotechnology, University of Naples Federico II, 80131 Naples, Italy; 2 CEINGE–Biotecnologie Avanzate Franco Salvatore, 80145 Naples, Italy; 3 Clinical Proteomics and Metabolomics Unit, School of Medicine and Surgery, University of Milano-Bicocca, Vedano al Lambro, 20900 Monza, Italy

The rapid and substantial advancements in proteomic and metabolomic technologies have revolutionized our ability to investigate biological systems. These modern techniques have become very popular due to their use in analyzing almost all cellular processes and functions which are crucial for normal cell activity such as protein interactions, metabolic processes, cell signaling and cellular reaction to stimuli [1,2,3,4]. Such progress enables the understanding of the molecular basis of disease and health and facilitates the identification of *potential* biomarkers and drug targets [5,6,7,8,9]. Nevertheless, it is appropriate to specify that many of the identified biomarkers are still in the early research stages and they lack sufficient validation for widespread clinical use.

The transformative impact of omics technologies in medical research and diagnostics has been unequivocally established, driving an unprecedented era of discovery over the past decade. A key advantage of these technologies is their ability to rapidly generate large volumes of data, enabling comprehensive analyses. For instance, proteomics has advanced our understanding of disease biomarkers, especially cancer, whilst metabolomics has emerged as a powerful tool for diagnosing inherited metabolic disorders [10,11]. Nowadays, each omics discipline has matured into a distinct branch of science, unlocking a wealth of applications and providing profound insights across diverse fields [12,13,14,15]. Additionally, the rapid evolution of artificial intelligence has significantly enhanced the ability to analyze large and complex datasets generated by omics sciences [16,17,18,19]. However, it is essential to recognize that the translation of omics data into actionable knowledge remains a significant challenge. Furthermore, while these approaches aim to minimize bias through high-throughput methodologies, they may overlook key complexities, such as the diversity of proteoforms, underscoring the need for continued refinement and integration with complementary analytical techniques.

Today, the integration of multi-omics data has become a cornerstone of advanced biomedical research. By harmonizing these datasets, researchers can unravel the molecular underpinnings of complex diseases and identify promising therapeutic targets, significantly amplifying our ability to tackle medical challenges with precision and innovation [20,21,22]. Mass spectrometry (MS)-based proteomic, metabolomic, and lipidomic technologies are leveraged to identify novel diagnostic and prognostic markers, unravel disease mechanisms, and uncover *potential* therapeutic targets, ultimately enabling more precise disease phenotyping [23,24,25]. However, while omics sciences are driving breakthroughs in biomedicine, many questions remain unresolved, particularly regarding proteoforms. Despite recent advancements [26], the full diversity of proteoforms, due to factors like alternative splicing and post-translational modifications, remains largely unexplored. This limits our understanding of proteome complexity and its role in disease onset and progression. The analysis of proteoforms from an omics perspective is crucial for clinical applications, making it essential to address it in future works. In our Topic ‘Proteomics and Metabolomics in Biomedicine, 2nd Volume’, we collected some of the most recent omics findings concerning the biomedical field, keeping the community updated as we expanded on our previous collection [27]. Interested readers will be informed of the current discoveries gathered thanks to the application of those cutting-edge technologies.

Proteomic and metabolomic approaches are extensively applied to study diverse human diseases, including cardiovascular damage, neurodegeneration and comorbidities of obesity. For instance, Sartore et al., adopting a targeted proteomic approach based on proximity extension assays, profiled the circulating plasma proteome of patients affected by type 2 diabetes. Their data revealed a panel of six plasma proteins that they proposed as promising predictive markers for the development of diabetes-related cardiovascular complications [28]. On a similar note, Songjang et al. performed a proteomic analysis of the vascular endothelial cell-derived damage-associated molecular patterns (DAMPs) in an in vitro model of early lipopolysaccharide-induced injury to identify *potential* damage-response proteins that could be used as biomarkers for the early diagnosis of sepsis [29].

There are several reasons why proteomics is the most appropriate technique for the analysis of human diseases, as shown in the review by Rusi and colleagues [30] where they discussed current research to examine the link between the olfactory system and neurodegenerative disorders from a proteoform point of view. Interestingly, most of the cited studies were based on liquid biopsy samples, thus highlighting the relevance of this minimally invasive modality for the derivation of biomolecular information as well as for the prognostic and therapeutic management of disease progression [31,32,33].

Other biomedical studies applying metabolomic technologies were also presented in our Topic. An untargeted metabolomic analysis was conducted on the serum of obese women patients with nonalcoholic steatohepatitis (NASH), identifying a unique metabolic lipid profile associated with HASH and possibly related with the physiopathology mechanism of that liver disease [34]. Another metabolomic work was carried out on the prefrontal cortex of rats exposed to chronic social isolation (CSIS), a model of depression, before and after fluoxetine treatment. The results provided a specific metabolic fingerprint capable of predicting depressive-like ongoing status, disease risk and treatment outcome [35].

Of note is the work of Shao et al. [36] that exploited a strategy of combining proteomics and metabolomics to elucidate the regulatory mechanism of diapause formation in the oriental armyworm. Specifically, they studied the meadow moth at different diapause stages and proposed the histone acetylation as a central epigenetic molecular marker of developmental decision in insects. These basic biological understandings, however, appeared fundamental when examining them from the One Health point of view. In fact, such findings highlight the importance of both the balance of interspecies coexistence and the need to control the spread of infectious organisms, emphasizing the interconnected health of humans, animals, and the environment [37,38,39]. From the same One Health perspective, the work of Kim et al. investigated the effects of zinc oxide nanoparticle (ZnO NP) exposure on kidney functionality. Through a lipidomic strategy, they demonstrated that ZnO NPs induce cytotoxicity in renal epithelial cells and modulate the production of lipid species [40].

Finally, advancements in MS methodologies and their applications in clinical practice have been explored, particularly in cancer therapy. Monitoring drug concentrations is vital to avoid interactions in combined treatments and personalize dosing. Robust analytical MS methods to measure drug levels in bodily fluids was urgently needed to ensure accurate checking and improve patient outcomes. Within this framework, Song et al. introduced an MS data-dependent acquisition mode integrated with a targeted MRM^HR^ workflow to enable the rapid and accurate quantification of the antitumoral agent camrelizumab in human serum [41]. Their approach underscores the significant clinical relevance of MS analysis and highlights its emerging role as a pivotal tool in biomedical research and practice.
